# Evaluation of Handheld Assays for the Detection of Ricin and Staphylococcal Enterotoxin B in Disinfected Waters

**DOI:** 10.1155/2011/132627

**Published:** 2011-07-14

**Authors:** Mary Margaret Wade, Tracey D. Biggs, Joseph M. Insalaco, Lisa K. Neuendorff, Vicky L. H. Bevilacqua, Amanda M. Schenning, Lisa M. Reilly, Saumil S. Shah, Edward K. Conley, Peter A. Emanuel, Alan W. Zulich

**Affiliations:** ^1^Edgewood Chemical Biological Center, U.S. Army, RDECOM, Edgewood Area, Aberdeen Proving Ground, MD 21010, USA; ^2^Science Applications International Corporation, P.O. Box 68, Gunpowder, MD 21010, USA; ^3^Nuclear, Biological and Chemical Contamination Avoidance, Edgewood Area, Aberdeen Proving Ground, MD 21010, USA

## Abstract

Development of a rapid field test is needed capable of determining if field supplies of water are safe to drink by the warfighter during a military operation. The present study sought to assess the effectiveness of handheld assays (HHAs) in detecting ricin and Staphylococcal Enterotoxin B (SEB) in water. Performance of HHAs was evaluated in formulated tap water with and without chlorine, reverse osmosis water (RO) with chlorine, and RO with bromine. Each matrix was prepared, spiked with ricin or SEB at multiple concentrations, and then loaded onto HHAs. HHAs were allowed to develop and then read visually. Limits of detection (LOD) were determined for all HHAs in each water type. Both ricin and SEB were detected by HHAs in formulated tap water at or below the suggested health effect levels of 455 ng/mL and 4.55 ng/mL, respectively. However, in brominated or chlorinated waters, LODs for SEB increased to approximately 2,500 ng/mL. LODs for ricin increased in chlorinated water, but still remained below the suggested health effect level. In brominated water, the LOD for ricin increased to approximately 2,500 ng/mL. In conclusion, the HHAs tested were less effective at detecting ricin and SEB in disinfected water, as currently configured.

## 1. Introduction

The protection of water supplies and water distribution infrastructure such as reservoirs is of great concern for those defending against bioterrorism. The US military is concerned because there exist multiple biological warfare agents (BWAs) that could be intentionally introduced into the water supply which could have serious implications for military operations as well as civilians located within a theater of operations. To protect potable water supplies, the Department of Defense (DoD) seeks to develop a rapid field test that would determine if field supplies of water are safe to drink by the warfighter during a military operation. The US armed services have fielded biological defense systems that are designed to protect against a large scale attack via BWAs dispersed through the air. These defensive systems utilize high-efficiency aerosol collectors that impinge the sampled air into a liquid buffer which is tested for BWAs using antibody-based handheld assays (HHAs). Civilian first responders use similar versions of these antibody-based HHAs for testing suspicious powder samples suspected to contain BWAs. Depending on which manufacturer of HHA is being tested, the technology can be low cost, requires no electrical power, and requires minimal to no sample preparation to test a sample. It was an HHA panel manufactured by the DoD that first identified *Bacillus anthracis* in the powder that spilled across a desk in Senator Daschle's office in the Hart Senate Office building in 2001. Three years later, a DoD HHA was again used to identify ricin toxin in the mail room of the Dirksen Senate Office building. These instances demonstrate that, when HHA technologies are used appropriately by an experienced operator trained to understand what the results mean, they can be a useful tool. Additionally, HHAs have proven fast and effective in the laboratory for the detection of *Francisella tularensis* [1] and biotoxins such as staphylococcal enterotoxin B (SEB) [2] and microcystins [3]. 

The DoD has initiated an effort called the Joint Chemical Biological and Radiological Agent Water Monitor (JCBRAWM) program to develop a system which will allow rapid field testing of drinking water supplies used by military personnel. The effectiveness of HHAs in detecting biological agents in the air and in powders has resulted in a desire to test these assays to gauge their effectiveness to protect drinking water supplies used by US warfighters. In the present study, HHAs from multiple manufacturers were evaluated for their effectiveness at detecting two specific targets, SEB and ricin, in multiple water matrices. The HHAs were not modified in any way to optimize for the detection of toxins within water supplies despite the fact that they were initially developed to test for concentrated agent collected from high-efficiency aerosol collectors and impinged into a phosphate buffered saline (PBS) solution rather than to test unbuffered water supplies. 

The ability of the HHAs to detect SEB and ricin in four different water matrices, representative of waters used by different branches of the armed forces, was evaluated. The four different water matrices were formulated tap water, formulated tap water with 1 mg/L free available chlorine (FAC), water treated by reverse osmosis (RO) with 1 mg/L FAC, and RO water with 2 mg/L total residual bromine. Suggested health effect levels as used by the US Military for ricin and SEB are 455 ng/mL and 4.55 ng/mL, respectively [4]. These minimal health effect levels were derived using the Tri Service Standards for field water and based on an average consumption of 10 liters per day for seven days for a 70 kg service member [4]. Therefore, detection of ricin and SEB in water at or below the suggested health effect level is desired.

## 2. Materials and Methods

### 2.1. Handheld Assays

Both singleplex (one-line) and multiplex (two-line and two-dot) HHA models produced by two manufacturers were evaluated in the present study for the detection of ricin toxin and SEB [5, 6].

### 2.2. Toxins

Active SEB (Toxin Technologies, Sarasota, Fla, USA) and active ricin whole toxin (Vector Laboratories, Burlington, ON, Canada) were obtained from the DoD's Critical Reagents Program (CRP) for use in the present study.

### 2.3. Formulated Tap Water Preparation

Formulated tap water was prepared following our previously published method [7]. Briefly, approximately 500 mL of ASTM Type I deionized water was added to a one-liter volumetric flask to which each stock solution was added to achieve the desired final concentration and the total volume was brought to one liter with deionized water. After 15–20 minutes, the pH was assessed and adjusted if needed (with minimal volume change) to 7.6–7.8. Once prepared, synthetic tap water was stored at 4°C with a shelf life of one week. 

### 2.4. Chlorine Stock Solution Preparation

Chlorine stock was prepared following our previously published method [7]. Briefly, one gram of calcium hypochlorite was added to twenty milliliters of ASTM Type I deionized water and shaken vigorously for one to two minutes. The solids were allowed to settle for five to ten minutes before using. The chlorine stock solution was used within eight hours.

### 2.5. Chlorine Addition

Chlorine was added following previously described methods [7]. Briefly, approximately five microliters of the chlorine stock solution (supernatant, not solids) was added to 100 mL of the formulated tap water or RO water, which was prepared by running local tap water through a commercial RO unit. The FAC was measured using a Hach DR 2500 spectrophotometer per manufacturer's instructions, and the FAC level was adjusted as needed to achieve the desired FAC level of 1 mg/L. Chlorinated water was considered stable for only one day and therefore made fresh daily just prior to use.

### 2.6. Bromine Addition

Bromine was added following previously described methods [7]. Briefly, 25 mL of bromine resin was transferred to a 500 mL, 0.20 *μ*m filter unit (Corning Life Science, Lowell, Mass, USA). RO water was poured into the same filter unit, filtered, and collected. The bromine resin was retained by the filter. Total residual bromine in the collected water was measured using a Hach DR 2500 spectrophotometer per manufacturer's instructions and adjusted as needed to achieve the desired final concentration of 2 mg/L.

### 2.7. Handheld Assay Testing

Each water type was prepared fresh daily as described above and spiked separately with each target at desired concentrations. After spiking, 100 *μ*L of sample was loaded onto the sample well of the HHA. The HHA was allowed to develop for 15 minutes prior to reading the result by eye. All HHAs were randomized prior to being read by a minimum of two people. Each challenge concentration was tested a minimum of two independent times with a minimum of three handheld assays per concentration. Limits of detection (LOD) were determined for each HHA type in each water matrix and defined as the minimum concentration of toxin that could be detected by eye. Presence of a line as observed visually by eye was considered a positive detection.

### 2.8. Circular Dichroism

Samples for circular dichroism (CD) were prepared by spiking RO water containing 1 mg/L FAC with SEB to a final concentration of 2 *μ*g/mL. Control samples received no chlorine. After 24 hours, sodium thiosulfate (0.005% w/v final concentration; Fisher Scientific Catalog number S446) was added to all samples to quench any remaining chlorine prior to analysis by CD. CD experiments employed a JASCO Model J-810 spectropolarimeter (JASCO Analytical Instruments, Easton, Md, USA) equipped with a PTC-423S Peltier thermoelectric temperature control system. CD spectra were obtained for SEB at 25°C from 260 to 190 nm with a data pitch of 0.5 nm, a band width of 1 nm, a response time of 1 sec, and a scanning speed of 10 nm/min. The CD was carried out on 2 *μ*g/mL samples in 1 cm quartz cuvettes and required long-time signal averaging. One hundred scans were collected and saved separately for each sample analyzed by CD. The concentration of SEB was verified for a representative stock solution by UV absorption at 277 nm using an E^1%^ for SEB of 14 [8]. Concentrations for the CD samples were calculated based on the dilution factors. For each sample, the blank-subtracted CD curve for 100 averaged scans was converted to molar ellipticity using a molecular weight of 28,366 daltons and fit with the Secondary Structure Estimation Application in JASCO's Spectra Manager (version 1.53.01) software to obtain the fraction of helix, beta sheet, turn, and random coil secondary structure. The percentages reported here were obtained using the Reed reference set for the secondary structure calculations [9].

### 2.9. Sodium Dodecyl Sulfate-Polyacrylamide Gel Electrophoresis (SDS-PAGE)

 Samples for SDS-PAGE were prepared by spiking RO water containing 1 mg/L FAC with SEB to a final concentration of 2 *μ*g/mL. Control samples received no chlorine. After one minute, one hour, and 24 hours, sodium thiosulfate (0.005% w/v final concentration) was added to all samples to quench any remaining chlorine prior to analysis by SDS-PAGE. Reducing gel eletrophoresis was carried out using the Mini-PROTEAN 2 Cell and 10–20% Tris-HCl PAGEr Gold Precast gels from Lonza (Rockland, Me, USA). Fifty nanograms of SEB samples and 1 : 20 diluted Mark12 unstained standard (Invitrogen) were prepared in SDS sample buffer, followed by heating at 95°C for 5 minutes prior to loading. Running buffer was prepared by dilution from 10x Tris/Glycine/SDS Buffer (BioRad). Electrophoresis was carried out at 120 volts for approximately 90 minutes. Following electrophoresis, the gel was fixed with 50% methanol and 7% acetic acid (both v/v) for a total of 30 minutes. The rapid staining protocol with SYPRO Ruby protein stain (Invitrogen) was used for the detection of SEB. The gel was washed with a solution of 10% methanol and 7% acetic acid (both v/v) for 30 minutes and imaged and documented with UV light using Syngene (Frederick, Md, USA).

## 3. Results

### 3.1. Performance in Disinfected Waters

All HHAs were evaluated for their performance in formulated tap water, formulated tap water with chlorine, RO water with chlorine, and RO water with bromine for the detection of ricin and SEB. Each water type was spiked with multiple concentrations of each toxin and loaded onto HHAs for testing until the LOD was determined for each HHA model. Resulting LODs for each model in each water are shown in Table 1. It was determined that ricin could be detected down to approximately 1 ng/mL for most HHA models in both formulated tap water and formulated tap with chlorine, while exhibiting only slightly higher LODs in RO with chlorine for most models. However, in RO with bromine, the LOD for ricin increased to 3,000 ng/mL for all models tested. For SEB, LODs in formulated tap water were low, ranging from 0.9 ng/mL to 12.5 ng/mL. However, in both chlorinated and brominated water, LODs increased to 2,500 ng/mL for singleplex HHAs and even higher to 5,000 or 10,000 ng/mL for multiplex models. In all cases, singleplex assays performed the same or better than multiplex assays. Detection below the suggested health effect level was desired; however, SEB was not detected below the suggested health effect level in chlorinated and brominated water and ricin was not detected below that level in brominated water.

### 3.2. Circular Dichroism of SEB

**Figure 1:**
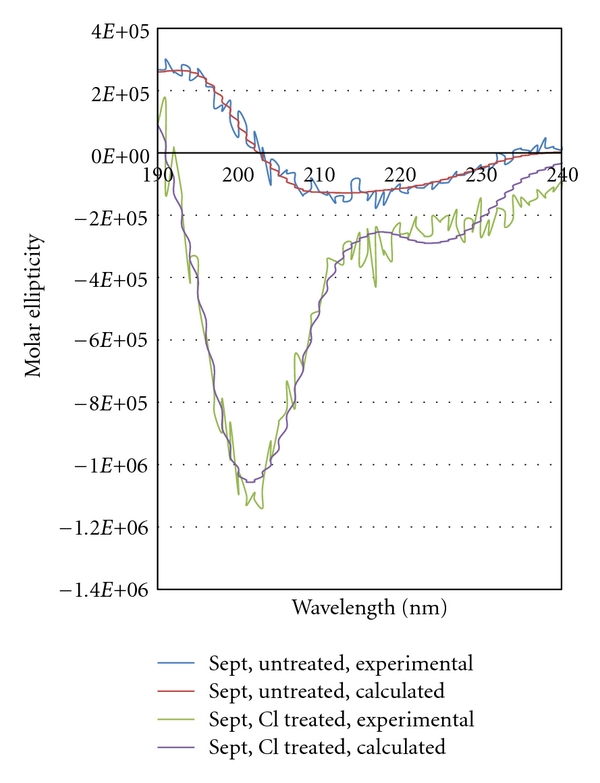
Overlay of the circular dichroism spectra for untreated (blue = experimental, red = curve fit) and chlorine-treated (green = experimental, purple = curve fit) SEB (2 *μ*g/mL). The initial free chlorine concentration for the treated sample was 1 mg/L. Each circular dichroism spectrum is the average of 100 blank-subtracted scans.

Due to the increased detection limits observed in chlorinated and brominated water, CD spectroscopy, which assesses the three-dimensional fold of a protein, was performed on samples of untreated SEB and SEB exposed to chlorine in an effort to evaluate the basis for increased LODs (Figure 1). For each sample, the individual CD scans were compared prior to averaging to ascertain the structural stability of the sample. The untreated SEB was stable throughout experimentation. Untreated SEB at 2 *μ*g/mL was determined to contain 14.3% helix, 34.50% sheet, 17.6% turn, and 33.6% random coil (rmsd < 0.1%). The CD spectrum of SEB treated with 1 mg/L FAC was stable over the time of experimentation. The curve-fitting calculation for chlorine-treated SEB resulted in 25.9% helix, 0.0% sheet, 0.0% turn, and 74.1 random coil (rmsd < 0.1%). 

### 3.3. SDS-PAGE

**Figure 2:**
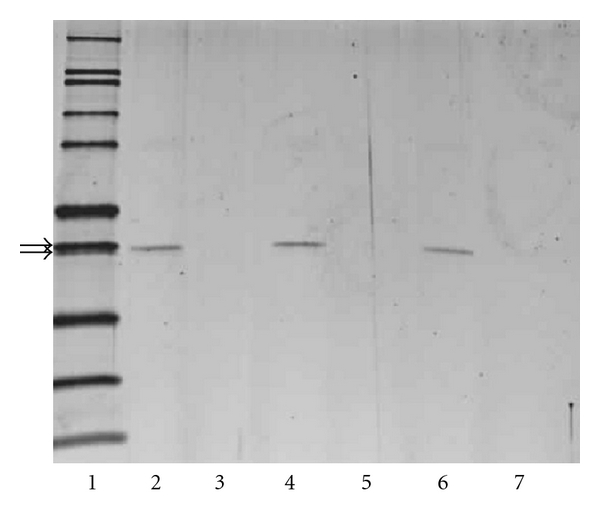
SDS-PAGE analysis of SEB treated with chlorine. Lane 1: molecular mass standards (in kilodaltons); lane 2: untreated control SEB after 1 min; lane 3: chlorine-treated SEB after 1 min; lane 4: untreated control SEB after 1 hour; lane 5: chlorine-treated SEB after 1 hour; lane 6: untreated control SEB after 24 hours; lane 7: chlorine-treated SEB after 24 hours. Arrows indicate 31 kDa molecular weight marker (top arrow) and 28 kDa SEB (bottom arrow).

SDS-PAGE was also performed to ascertain the overall effect of chemical reaction with chlorine on the molecular weight of SEB. For the treated and untreated SEB, 1.5 *μ*g of SEB was loaded per well. As shown in Figure 2, for untreated SEB, the SEB was visible on the gel as a band with a molecular weight of approximately 28 kDa for all time points tested. For chlorine-treated SEB, no band was visible at the same molecular weight as untreated SEB for any of the time points tested. No other bands were visible for the chlorine-treated SEB. 

## 4. Discussion

Ricin and SEB are considered potential biological threat agents as both can cause illness either by inhalation or ingestion. SEB could potentially be used to attack low-volume water supplies; however, due to the fact that ricin is less toxic by oral ingestion, it may be a less likely threat to drinking water [10]. However, detection of both agents in water is still a concern and technologies capable of detection in water must be investigated. One such potential technology is the use of HHAs for detection in water. Performance of HHAs for air and surfaces is well known; however, the use of HHAs for detection of agents in water is less recognized. Therefore, the application of HHAs for detection in water was assessed in this study.

In formulated tap water, detection of SEB and ricin was achieved at or below the suggested health effect levels for each model of HHA in almost every case. However, the presence of disinfectants proved problematic with regard to detecting either SEB or ricin using all models of HHAs. The increased LODs observed when disinfectants were present are believed to be due to the degradation of the target or toxins by chlorine and bromine. Both chlorine and bromine are very reactive elements capable of reacting with many substances. Both disinfectants are extremely effective sanitizers, killing microbes in minutes. Therefore, it is likely that protein toxins would also be affected by these strong oxidants. In fact, sodium hypochlorite is known to completely inactivate SEB and ricin toxin [11, 12]. Therefore, when exposed to chlorine or bromine, protein toxins could become damaged or denatured causing antibodies present in an antibody-based assay, such as HHAs, to no longer bind to the altered antigen as effectively. This decrease in binding efficiency could yield higher LODs for the target, such as those observed in the present evaluation. 

To address this issue, CD spectroscopy and SDS-PAGE were employed to study the effect of chlorine treatment on the structure of SEB. The CD results for untreated SEB were similar to those obtained by Singh et al. for SEB at 0.5-0.6 mg/mL in 10 mM sodium phosphate [13]. The CD results for chlorine-treated SEB indicate a significant change in three-dimensional fold resulting in less well-defined structure for the chlorine-treated SEB relative to the untreated SEB. Based on curve-fitting calculations, the percentage of random coil more than doubled upon treatment with chlorine. The secondary structure percentages obtained from the CD curve-fitting routine may well represent an average of multiple SEB-based species in solution, which would be consistent with the SDS-PAGE results. The absence of bands for the chlorine-treated SEB could occur if the SEB was cleaved into smaller peptides that diffuse out of the gel or if multiple higher molecular weight species are formed, with no single species being concentrated enough to be visualized on the gel. In either of these cases, chiral components would be present in the sample and would lead to a measureable CD spectrum. The CD and PAGE results together suggest a major change in SEB structure that may result in multiple SEB-based antigens, the majority of which are less easily recognized and not bound as effectively as untreated SEB by the antibody present on the membrane of the assay. The decreased binding capacity for the SEB-based antigen(s) would account for the increased detection limits seen for SEB in chlorinated water. These results support the idea that increased LODs in disinfected water are caused by disruption in antigen-antibody binding due to degradation of the antigen. 

In addition to the detection studies reported herein, currently studies are being performed to address whether both ricin and SEB remain active, or toxic, after exposure to chlorine and bromine. However, regardless of whether activity remains, the ability to detect the presence of each toxin in disinfected water supplies is important. Presence of these agents could indicate a potential compromise or attack on the water supply, thereby revealing security risks as well as potential health risks. Additionally, it was noted in the present study that chlorine did not impact detection levels of ricin as significantly as detection levels of SEB under the test conditions employed. Detection with HHAs was performed immediately after spiking each water with toxin. Additional exposure time may be required in order for ricin detection to be affected in the same manner as that observed for SEB in chlorinated water. This could be due to a difference in protein structure or stability in chlorinated water between the two toxins. Further tests allowing for prolonged incubation of ricin in chlorinated water prior to detection could address this issue.

## Figures and Tables

**Table 1:** LODs^a^ or ricin and SEB in multiple water matrices using handheld assays.

	Manufacturer 1		Manufacturer 2		
	1-line	2-line	1-line	2-line	2-dot
Ricin LODs					

Tap	1.1	7.1	1.2	1.3	5.9
Tap + Cl	1.8	16.7	1.0	1.3	1.3
RO + Cl	3.3	56.9	2.2	2.5	4.9
RO + Br	3000	3000	3000	3000	3000

SEB LODs					

Tap	3.1	12.5	0.9	1.1	1.9
Tap + Cl	2500	5000	2500	5000	5000
RO + Cl	2500	10000	2500	5000	5000
RO + Br	2500	5000	2500	5000	5000

^a^All LODs reported are in ng/mL.

Concentrations were tested a minimum of two independent times with a minimum of three handheld assays per concentration.
